# An Innovative Field-Applicable Molecular Test to Diagnose Cutaneous *Leishmania Viannia spp*. Infections

**DOI:** 10.1371/journal.pntd.0004638

**Published:** 2016-04-26

**Authors:** Omar A. Saldarriaga, Alejandro Castellanos-Gonzalez, Renato Porrozzi, Gerald C. Baldeviano, Andrés G. Lescano, Maxy B. de Los Santos, Olga L. Fernandez, Nancy G. Saravia, Erika Costa, Peter C. Melby, Bruno L. Travi

**Affiliations:** 1 Division of Infectious Diseases, Department of Internal Medicine, University of Texas Medical Branch (UTMB), Galveston, Texas, United States of America; 2 Center for Tropical Diseases (CTD), University of Texas Medical Branch (UTMB), Galveston, Texas, United States of America; 3 Laboratório de Pesquisa em Leishmaniose, Instituto Oswaldo Cruz, Rio de Janeiro, Brazil; 4 U.S. Naval Medical Research Unit No. 6 (NAMRU-6), Lima, Perú; 5 Facultad de Salud Pública y Administración, Universidad Peruana Cayetano Heredia, Lima, Peru; 6 Centro Internacional de Entrenamiento e Investigaciones Médicas (CIDEIM), Cali, Colombia; 7 Department of Microbiology and Immunology, University of Texas Medical Branch (UTMB), Galveston, Texas, United States of America; Universidade Federal de Minas Gerais, BRAZIL

## Abstract

Cutaneous and mucosal leishmaniasis is widely distributed in Central and South America. *Leishmania of the Viannia* subgenus are the most frequent species infecting humans. *L*. *(V*.*) braziliensis*, *L*. *(V*.*) panamensis* are also responsible for metastatic mucosal leishmaniasis. Conventional or real time PCR is a more sensitive diagnostic test than microscopy, but the cost and requirement for infrastructure and trained personnel makes it impractical in most endemic regions. Primary health systems need a sensitive and specific point of care (POC) diagnostic tool. We developed a novel POC molecular diagnostic test for cutaneous leishmaniasis caused by *Leishmania (Viannia)* spp. Parasite DNA was amplified using isothermal Recombinase Polymerase Amplification (RPA) with primers and probes that targeted the kinetoplast DNA. The amplification product was detected by naked eye with a lateral flow (LF) immunochromatographic strip. The RPA-LF had an analytical sensitivity equivalent to 0.1 parasites per reaction. The test amplified the principal *L*. *Viannia* species from multiple countries: *L*. *(V*.*) braziliensis* (n = 33), *L*. *(V*.*) guyanensis* (n = 17), *L*. *(V*.*) panamensis* (n = 9). The less common *L*. *(V*.*) lainsoni*, *L*. *(V*.*) shawi*, and *L*. *(V*.*) naiffi* were also amplified. No amplification was observed in parasites of the *L*. *(Leishmania)* subgenus. In a small number of clinical samples (n = 13) we found 100% agreement between PCR and RPA-LF. The high analytical sensitivity and clinical validation indicate the test could improve the efficiency of diagnosis, especially in chronic lesions with submicroscopic parasite burdens. Field implementation of the RPA-LF test could contribute to management and control of cutaneous and mucosal leishmaniasis.

## Author Summary

Cutaneous leishmaniasis is a parasitic disease transmitted by the bite of sandflies that produces skin ulcers. The severe, disfiguring form of the disease is characterized by parasite dissemination to the mucosa of the nose and palate. Current diagnosis is based on microscopy which has low sensitivity in chronic cases. Molecular methods (PCR) that detect parasite DNA are highly sensitive but costs and personnel training make it impossible to implement it in resource-limited areas. We developed a novel molecular method (RPA-LF) that could be applied in the field because it does not require sophisticated equipment. It is also very sensitive and specific to detect the principal *Leishmania* species that produce cutaneous leishmaniasis in Latin America. Future field implementation of RPA-LF could have a positive impact on disease management and control.

## Introduction

Dermal and mucosal leishmaniasis are widely distributed in Central and South America, affecting an estimated 190,000–300,000 people annually [1]. Many different *Leishmania* species grouped under the subgenera *Leishmania* or *Viannia* can produce dermal leishmaniasis. Epidemiologically, *Viannia* is the most relevant subgenus in this region since it is highly prevalent and also responsible for metastatic mucosal leishmaniasis (*L*. *(V*.*) braziliensis*, *L*. *(V*.*) panamensis*, *L*. *(V*.*) guyanensis*), the severe form of tegumentary disease [2,3].

Microscopy is still the most common diagnostic method used in endemic regions but its sensitivity is not ideal and markedly affected by the experience of the microscopist [4]. Furthermore, the sensitivity of this method tends to decrease with disease chronicity, which is characterized by a low number of amastigotes in the lesions [4]. Serological tests were used in the past and the identification of new antigens and formats for serodiagnosis of American cutaneous leishmaniasis is still considered [5,6]. However, in general, they have proven to be of limited value due to the variable immune responses of patients and no clear distinction between current disease and past infections or exposure.

Conventional or quantitative PCR from dermal or mucosal samples have high diagnostic sensitivity (≈87–98%) and specificity (≥84%) [7]. This molecular method is currently the gold standard in leishmaniasis reference centers or tertiary care facilities. However, the need for expensive equipment, trained personnel, and relatively complex laboratory facilities are beyond the capability of the typical health infrastructure in endemic areas.

Therefore, there is a clear need to provide primary health systems with diagnostic tools that are simple, easy to use and have good sensitivity and specificity. To address this critical gap, we developed a novel-point-of-care molecular test to diagnose dermal and mucosal leishmaniasis produced by *Leishmania Viannia* spp. We designed primers and probes that targeted the kinetoplast DNA minicircles, similar to the strategy we used previously to detect *L*. *infantum chagasi* [8]. Leishmania DNA was amplified using isothermal Recombinase Polymerase Amplification (RPA), a method originally described by Piepenburg et al.[9]. The amplification product was detected in a lateral flow immunochromatographic strip (LF) which is read with the naked eye. Its analytical sensitivity and specificity indicated that it could be used as a point-of-care diagnostic test for dermal and mucosal leishmaniasis in endemic areas of Latin America.

## Materials and Methods

### Ethical statement

The current study was approved by the Office of Sponsored Programs of the University of Texas Medical Branch. The activities of NAMRU-6 were conducted in compliance with all applicable federal and international regulations governing the protection of human subjects. This study was approved by the Institutional Review Board of the U.S. Naval Medical Research Unit 6 (NMRCD.2007.0018), and administratively approved by the Madre de Dios Regional Health Directorate in Peru. CIDEIM provided DNA samples stored in its cryobank and consent forms from patients for multiple uses were obtained. The IRB of UTMB waived ethical approval for this study based on the utilization of DNA from de-identified patients.

### Design of primers and probe

The primer sets for *Leishmania Viannia* are 30–35 nucleotides long and target conserved sequences identified by computational alignment of *L*. *Viannia* kDNA minicircle sequences reported in GenBank. Primers were designed with 40–60% GC content, few direct/inverted repeats, and absence of long homopolymer tracts. We focused principally on conserved regions and to a lesser extent on regions with moderate variability, obtaining a 120 bp RPA amplicon in agarose gels. To enable detection by lateral flow, the reverse primer was biotinylated at the 5’ end. We designed a 45bp conserved internal probe (Biosearch technologies -Petaluma, CA) that included FAM (5’-carboxy fluorescein amidite) at the 5’ end, an internal dSpacer and a SpacerC3 in the 3’ end, as suggested by the manufacturer (TwistDx). Forward Primer: *Fw- GATGAAAATGTACTCCCCGACATGCCTCTG*. Reverse Primer: *Rev-bio-CTAATTGTGCACGGGGAGGCCAAAAATAGCGA*. Internal Probe: The probe contains a 5’-fluorescein group (FAM), an internal (THF)-tetrahydrofuran residue, and a C3 spacer block at the 3’ end. *Probe-****FAM****-GTAGGGGNGTTCTGCGAAAACCGAAAAATG[THF]CATACAGAAACCCCG[C3-spacer]*.

### Parasite DNA isolation

Promastigote suspensions of reference strains or clinical strains thawed from cryopreserved stocks or absorbed in Whatman FTA filter paper (Sigma-Aldrich) were subjected to 95°C for 2 minutes in a dry bath to lyse the parasites. DNA purification for the majority of the samples was carried out using the DNeasy Blood & Tissue Kit (Qiagen) following the recommendations of the vendor and adjusted to10 ng/μL. In addition, a small number of clinical samples (n = 8) obtained from ulcers of Peruvian patients suspected of having cutaneous leishmaniasis were absorbed in Whatman FTA filter paper. The filter papers (n = 2 of 6 mm diameter) were placed in direct contact with the ulcer allowing lymph and cells to be absorbed by the papers. Once dried, each patient sample was packed individually using sealed plastic bags which were labeled and transported to the central lab at room temperature. Two 3mm diameter filter papers were punched from the original samples, washed 3 times with 200 μL of FTA buffer (Whatman, GE Healthcare Life Sciences) followed by 3 washes with TE buffer pH 8 (Sigma). The papers were then suspended in water and heated at 95°C for 30 minutes; 2.5 μL of the solution were used to run the RPA-LF test.

### RPA reaction and lateral flow reading

The amplification mixture was comprised of: 1) forward primer, 2) biotinylated reverse primer, 3) FAM-labeled probe (stocks-5μM), 4) magnesium acetate, and 5) the rehydrated cocktail (Twist amp nfo RPA kit -TwistDx, UK). Parasite DNA (5–25ng/μL) was immediately added to the mixture and subjected to amplification at 45°C for 30 minutes using a dry bath. The RPA product was diluted 1:25 in the dipstick assay buffer and 30 μL were placed in a 1.5 Eppendorf tube or 96-well microplate. The bottom tip of the lateral flow strip was then immersed in the sample (GenLine HybriDetect, Milenia Biotec, Germany) making the amplification product run upwards by capillarity. Parasite amplification was confirmed with the naked eye after 5 minutes by the appearance of the test band in the lower part of the strip. This band is produced when anti-biotin antibodies immobilize the amplified DNA which contains the biotinylated primers. The gold particles, which are covered with mouse anti-FAM antibodies, bind to the probe labeled with FAM making the test band visible. The reaction was validated by the appearance of the control band in the upper part of the strip. This band appears upon the immobilization of excess free-gold particles (which are covered with mouse antibodies) by means of anti-mouse antibodies.

### Quantitative PCR

The RPA-LF sensitivity was compared with SYBRgreen real-time PCR using the primers described by Pita-Pereira *et al*. [10].

### *Leishmania* samples

The analytical evaluations of RPA-LF were carried out using known concentrations of DNA (10ng/μL). We evaluated banked strains of *L*. *braziliensis* from Brazil (n = 15), Colombia (n = 5), and Peru (n = 13); *L*. *guyanensis* from Brazil (n = 11) and Colombia (n = 6); *L*. *panamensis* from Colombia (n = 7), Nicaragua (n = 1), and Panama (n = 1); *L*. *lainsoni* from Brazil (n = 3) and Peru (n = 7); and *L*. *shawi* (n = 2) and *L*. *naiffi* (n = 6) from Brazil. Also, we evaluated DNA purified from lesion biopsies of patients from Peru who were infected with *L*. *braziliensis* (n = 9) and *L*. *guyanensis* (n = 4), as well as non-leishmanial (PCR-negative) skin lesions (n = 5).

## Results

**Fig 1:**
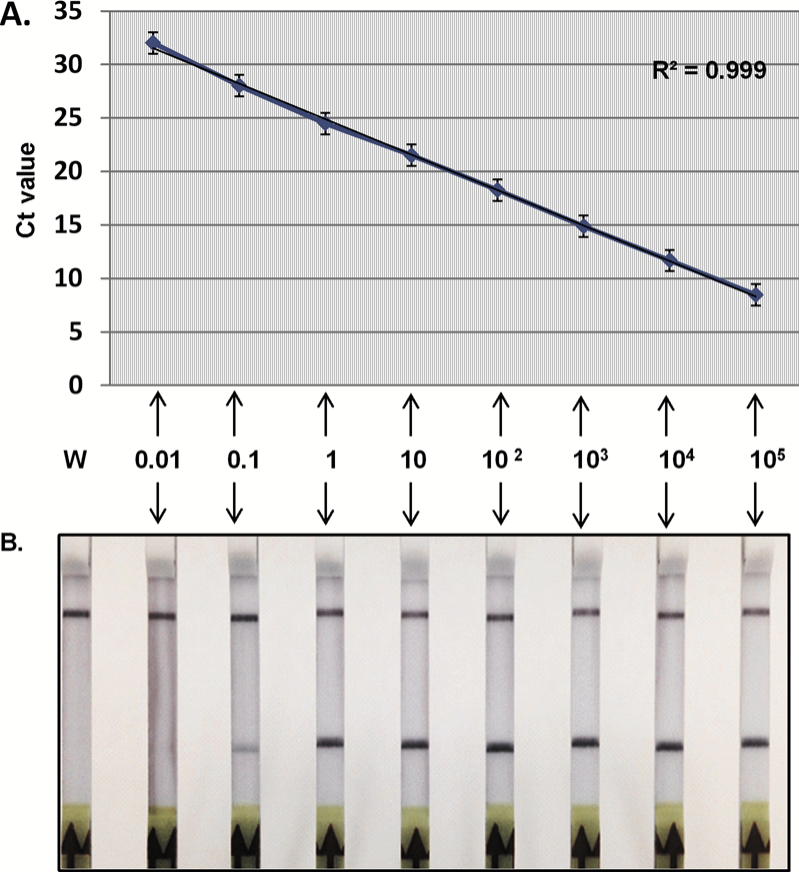
Sensitivity of RPA-LF to detect *L*. *viannia spp*. compared with real-time PCR (the current gold standard). Ten-fold serial dilutions of parasite DNA, extracted with Qiagen DNeasy blood and tissue kit, were amplified by qPCR (SYBRgreen) (A) or RPA-LF (B). W = water. The control band is the upper band, while the test band is the lower band.

**Fig 2:**
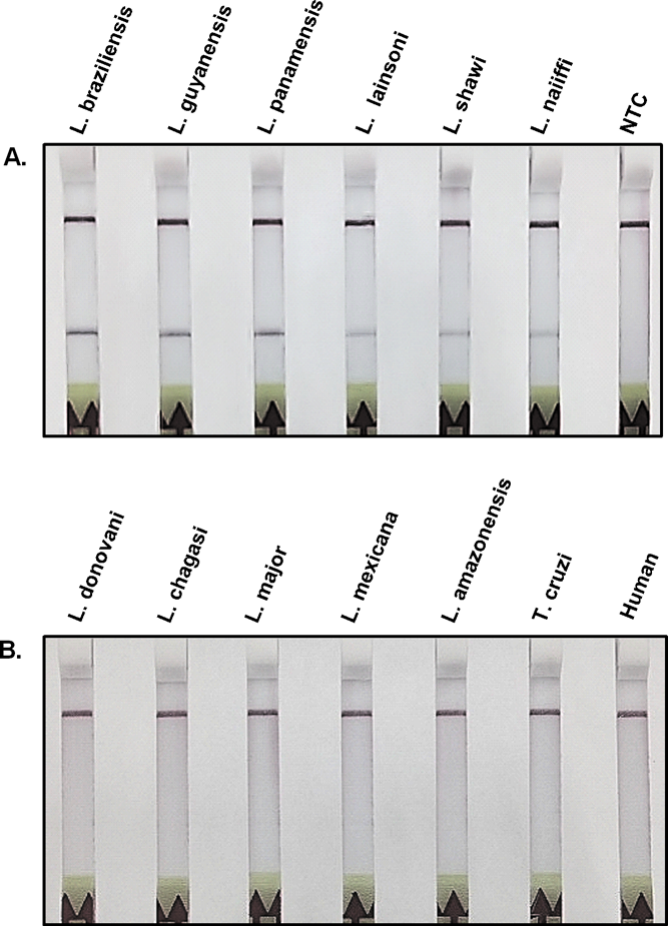
Specificity of RPA-LF to amplify species of the *Viannia* subgenus. A) The most relevant *L*. *Viannia* species (*L*. *braziliensis*, *L*. *guyanensis*, *L*. *panamensis*) produced stronger bands in the lateral flow strip than other less common species of this subgenus. B) Species of the *Leishmania* subgenus, *Trypanosoma cruzi*, and human DNA were not amplified by the RPA-LF test. NTC = no template control.

The RPA-LF amplified *Leishmania* DNA with an analytical sensitivity equivalent to 0.1 parasite per reaction, which corresponded to aCt value of 28 in the real-time PCR used as the gold standard (**Fig 1**). The capacity of RPA-LF to detect the most relevant species of the subgenus *Viannia* was initially determined by the amplification of a small number of banked strains of *Leishmania Viannia* spp: *L*. *braziliensis*, *L*. *panamensis*, *L*. *guyanensis*, *L*. *lainsoni*, *L*. *shawi* and *L*. *naiffi*. The specificity was confirmed by the lack of amplification of *L*. *donovani*, *L*. *chagasi*, *L*. *mexicana*, *L*. *amazonensis*, *L*. *major*, *Trypanosoma cruzi* and human DNA (**Fig 2**).

We further evaluated panels of strains from different species within the *Viannia* subgenus isolated in endemic areas of Brazil, Colombia, and Peru. Fifteen out of 15 *L*. *braziliensis* strains from Brazil, 6/6 strains from Colombia, and 12/12 from Peru, isolated from humans or dogs from different geographical areas, were amplified by RPA-LF **(Table 1)**. The test also demonstrated good sensitivity to detect several *L*. *guyanensis* strains obtained from endemic regions of Brazil (11/11) and Colombia (6/6) **(Table 1)**. Similarly, *L*. *panamensis* strains originally isolated from patients of Colombia (7/7), Nicaragua (1/1), and Panama (1/1) were readily amplified by RPA-LF. A small group of *L*. *Viannia* species known to occasionally infect humans were also evaluated by RPA-LF. Two Brazilian strains of *L*. *shawi*, a species closely related to *L*. *guyanensis*, produced strong bands indicating that the primers efficiently amplified this parasite species. However, 5/6 strains of *Leishmania naiffi*, usually found in mammals of the Amazon region and less frequently in other parts of South America, were amplified less efficiently than other *Viannia* species and generated weaker bands **(Table 1)**. In the case of *L*. *lainsoni*, a parasite found in wild mammals and sporadically infecting humans, RPA-LF produced a weak yet clearly detectable band in 3/3 strains from Brazil and 6/7 from Peru. One *L*. *naiffi*-*L*. *lainsoni* hybrid from Brazil was also detected by RPA/LF. Collectively, these results indicated that the test is capable of detecting all the epidemiologically relevant species of the *Viannia* subgenus. We developed an interactive map that depicts the geographical distribution of *Leishmania* species evaluated by RPA-LF (http://www.scribblemaps.com/maps/view/Leish_Viannia/9-18-15).

**Table 1:** RPA-LF detection of *Leishmania Viannia* species isolated from different countries in Latin America.

*Leishmania* spp.	Country	Region^1^	WHO code	RPA-LF	Map code^2^
***L*. *braziliensis***	**Brazil**	Pará	MHOM/BR/1975/M2903	+	**1**
		Ceará	MHOM/BR/1987/H-210	+	**2**
		Amazonas	MHOM/BR/1988/IM3482	+	**3**
		Ceará	MCAN/BR/1990/C35	+	**4**
		Ceará	MCAN/BR/1991/C51	+	**5**
		Amazonas	MHOM/BR/1994/IM3946	+	**6**
		Espírito Santo	MHOM/BR/1994/HAD-1	+	**7**
		Bahia	MHOM/BR/1996/SBS	+	**8**
		Bahia	MHOM/BR/2001/LTCP13183	+	**9**
		Acre	MHOM/BR/2002/NMT-RBO037	+	**10**
		Bahia	MHOM/BR/2001/NMT-LTCP14369-P	+	**11**
		Rio de Janeiro	MHOM/BR/2008/NC	+	**12**
		Pernambuco	MHOM/BR/2010/MMS	+	**13**
		Santa Catarina	MHOM/BR/2006/LSC128	+	**14**
		Santa Catarina	MHOM/BR/2006/LSC185	+	**15**
	**Colombia**	Caqueta	MHOM/CO/87/1270	+	**16**
		Nariño	MHOM/CO/85/2388	+	**17**
		Putumayo	MHOM/CO/82/L71	+	**18**
		Caqueta	MHOM/CO/88/1403	+	**19**
		Meta	MHOM/CO/85/1110	+	**20**
		Nariño	MHOM/CO/97/3144	+	**21**
	**Peru**	Cusco	MHOM/PE/14/LDP-0053	+	**22**
		Loreto	MHOM/PE/14/LDP-0057	+	**23**
		Junín	MHOM/PE/14/LDP-0060	+	**24**
		Junín	MHOM/PE/14/LDP-0065	+	**25**
		Cusco	MHOM/PE/14/LDP-0067	+	**26**
		Junín	MHOM/PE/14/LDP-0073	+	**27**
		Cusco	MHOM/PE/14/LDP-0075	w	**28**
		Madre de Dios	MHOM/PE/13/LDP-2036	+	**29**
		Madre de Dios	MHOM/PE/13/LDP-2039	+	**30**
		Madre de Dios	MHOM/PE/13/LDP-2059	+	**31**
		Madre de Dios	MHOM/PE/14/LDP-2074	+	**32**
		WHO	MHOM/PE/84/LTB300	+	**33**
***L*. *guyanensis***	**Brazil**	Amazonas	MHOM/BR/1997/NMT-MAO 210P	+	**34**
		Amazonas	MHOM/BR/1997/NMT-MAO 212P	+	**35**
		Amazonas	MHOM/BR/1997/NMT-MAO 237P	+	**36**
		Amazonas	MHOM/BR/1997/NMT-MAO 246P	+	**37**
		Amazonas	MHOM/BR/1997/NMT-MAO 292P	+	**38**
		Amazonas	MHOM/BR/1997/NMT-MAO 307P	+	**39**
		Amazonas	MHOM/BR/1997/NMT-MAO 317P	+	**40**
		Amazonas	MHOM/BR/1997/NMT-MAO 325P	+	**41**
		Amazonas	MHOM/BR/2007/031-LOP	+	**42**
		Amazonas	MHOM/BR/2007/033-MECM	+	**43**
		WHO	MHOM/BR/75/M4147	+	**44**
	**Colombia**	Caqueta	MHOM/CO/83/1028	+	**45**
		Putumayo	MHOM/CO/82/L76	+	**46**
		Putumayo	MHOM/CO/82/L75	+	**47**
		Caqueta	MHOM/CO/88/1390	+	**48**
		Tolima	MHOM/CO/2008/A197	+	**49**
		Putumayo	MHOM/CO/83/1011	+	**50**
***L*. *panamensis***	**Nicaragua**	Chontales	MHOM/NI/1988/XD45	+	**51**
	**Colombia**	Putumayo	MHOM/CO/92/1735	+	**52**
		Valle	MHOM/CO/84/1048	+	**53**
		Nariño	MHOM/CO/85/2476	+	**54**
		Nariño	MHOM/CO/85/2472	+	**55**
		Cauca	MHOM/CO/86/1180	+	**56**
		Narino	MHOM/CO/83/2017	+	**57**
		Cauca	MHOM/CO/95/1989	+	**58**
	**Panama**	WHO	MHOM/PA/71/LS94	+	**59**
***L*. *lainsoni***	**Brazil**	Pará	MHOM/BR/1981/M6426	+	**60**
		Rondônia	MCOE/BR/1983/IM1367	+	**61**
		Pará	MCUN/BR/1983/IM1721	+	**62**
	**Peru**	Amazonas	MHOM/PE/14/LDP-0061	w	**63**
		Loreto	MHOM/PE/13/LDP-1021	+	**64**
		Madre de Dios	MHOM/PE/15/LDP-2138	+	**65**
		Madre de Dios	MHOM/PE/15/LDP-2169	w	**66**
		Madre de Dios	MHOM/PE/15/LDP-2236	+	**67**
		Madre de Dios	MHOM/PE/15/LDP-2242	-	**68**
		Huanuco	MHOM/PE/88/BAB1730	+	**69**
***L*. *shawi***	**Brazil**	Pará	IWHI/BR/1985/IM2322	+	**70**
		Pará	MCEB/BR/1984/M8408	+	**71**
***L*. *naiffi***	**Brazil**	Pará	ISQU/BR/1985/IM2264	+	**72**
		Pará	MDAS/BR/1987/IM3280	-	**73**
		Pará	MDAS/BR/1979/M5533	+	**74**
		Amazonas	MHOM/BR/1991/IM3740	+	**75**
		Pará	MHOM/BR/2011/S50	+	**76**
		Pará	MHOM/BR/2011/58-AMS	w	**77**
***L*. *naiffi/ lainsoni***		Acre	MHOM/BR/2002/NMT-RBO004	+	**78**

+ indicates positive reading; W = indicates weak band

^1^Region: Brazil = State; Colombia = Department; Peru = Region

^2^Link to the interactive map: http://www.scribblemaps.com/maps/view/Leish_Viannia/9-18-15

In a small number of clinical samples we found that RPA-LF has excellent agreement with PCR as determined in DNA samples from patients of Peru infected with *L*. *braziliensis* or *L*. *guyanensis* **(Table 2)**. All 9 of the samples from clinical lesions due to *L*. *(V*.*) braziliensis*, and all 4 of the samples from clinical lesions due to L. (*V*.*) guyanensis* were positive by RPA-LF. The samples from negative controls were uniformly negative by RPA-LF. The high sensitivity and specificity identified with these limited number of samples warrants large-scale field testing to determine the diagnostic sensitivity of the RPA-LF.

**Table 2:** Agreement between RPA-LF and PCR to amplify *Leishmania Viannia* DNA purified from lesions of cutaneous leishmaniasis patients from Peru.

*Leishmania* spp.	Country	Region	WHO Code	qPCR	RPA-LF	Map Code^1^
***L*. *braziliensis***	**Peru**	Ucayali	MHOM/PE/2012/LDP-0005-Bx	*L*.*b*.	+	**79**
		Cusco	MHOM/PE/2012/LDP-0011-Bx	*L*.*b*.	+	**80**
		Junín	MHOM/PE/2012/LDP-0012-Bx	*L*.*b*.	+	**81**
		Madre de Dios	MHOM/PE/2013/LDP-0034-Bx	*L*.*b*.	+	**82**
		Junín	MHOM/PE/2014/LDP-0052-Bx	*L*.*b*.	+	**83**
		Junín	MHOM/PE/2014/LDP-0052-FP	*L*.*b*.	+	**84**
		Loreto	MHOM/PE/2014/LDP-0057-Bx	*L*.*b*.	+	**85**
		Loreto	MHOM/PE/2014/LDP-0057-FP	*L*.*b*.	+	**86**
		Loreto	MHOM/PE/2014/LDP-0057-L	*L*.*b*.	+	**87**
***L*. *guyanensis***		Huánuco	MHOM/PE/2012/LDP-0007-Bx	*L*.*g*.	+	**88**
		San Martin	MHOM/PE/2012/LDP-0014-Bx	*L*.*g*.	+	**89**
		San Martin	MHOM/PE/2013/LDP-0041-Bx	*L*.*g*.	+	**90**
		San Martin	MHOM/PE/2013/LDP-0041-FP	*L*.*g*.	+	**91**
**Negative Controls**		San Martin	MHOM/PE/2012/LDP-0017-Bx	-	-	
		Junín	MHOM/PE/2012/LDP-0030-Bx	-	-	
		Ucayali	MHOM/PE/2013/LDP-0042-Bx	-	-	
		Cusco	MHOM/PE/2013/LDP-0043-Bx	-	-	
		Iquitos	MHOM/PE/2015/LDP-0083-Bx	-	-	

+ indicates positive reading

*L*.*b*. = *L*. *braziliensis* as determined by qPCR; *L*.*g*. = *L*. *guyanensis* as determined by qPCR.

^1^Link to the interactive map: http://www.scribblemaps.com/maps/view/Leish_Viannia/9-18-15

**Fig 3:**
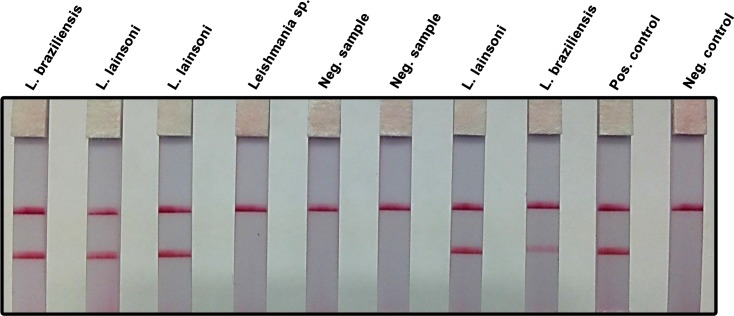
RPA-LF amplification of clinical samples using a simplified DNA extraction method. Samples from patients suspected of having cutaneous leishmaniasis were obtained by pressing Whatman FTA filter paper (two 6 mm diameter discs) over the dermal lesions. Two, 3 mm diameter papers were cut from the original samples using a punch, washed thrice with FTA washing reagent and twice with TE buffer pH 8. A 2.5 μL aliquot was amplified by RPA and subsequently read using a lateral flow strip. Patients infected with *Leishmania Viannia* spp. (*L*. *lainsoni*, *L*. *braziliensis*) were readily detected. The test did not amplify a strain originally labeled as *Leishmania* sp. in NAMRU-6, Peru. Presumably, it did not belong to the *Viannia* subgenus as confirmed by real-time PCR at UTMB. There was agreement between NAMRU-6 and UTMB labs with regard to the negative clinical samples. *L*. *lainsoni* was used as positive control; the negative control was run without template.

A potential limitation for field application of molecular diagnostic methods is the need for equipment such as vortex and high speed centrifuge to purify DNA from the clinical sample. We evaluated a DNA extraction method based on brief washing, elution and 95°C heating of the sample absorbed in filter paper (details in Materials & Methods). Using this approach we successfully amplified by RPA-LF all the samples of patients infected with *Leishmania Viannia* spp. as confirmed by real time PCR (**Fig 3**). This result indicated that RPA-LF could be implemented in basic diagnostic settings.

## Discussion

We developed a field-applicable molecular diagnostic test that distinguishes between the subgenera *Viannia* and *Leishmania* by selectively detecting strains of the *Viannia* subgenus. Our primers and probes were designed to target the kinetoplast DNA minicircles due to the high copy number (≈ 10,000) of this circular network of genomic mitochondrial DNA [11]. This remarkable number of copies provides a comparative advantage over other parasite targets with regard to test sensitivity. We targeted the *Viannia* subgenus because it encompasses the most relevant species causing cutaneous leishmaniasis in Latin America. The evaluation of the RPA-LF test included strains from Brazil, Colombia, and Peru, in which the recently reported incidence was 26,008, 17,420, and 6,405 cases/year, respectively [1]. The number of patients requiring diagnosis in these countries could be even greater since it was estimated that under-reporting varied between 2.8 and 4.6 fold [1].

The discrimination between *Viannia* and *Leishmania* subgenera is clinically relevant because in Latin America these infections may be treated differently [12]. Also, infection with *L*. *braziliensis*, *L*. *panamensis*, and less frequently *L*. *guyanensis* require prolonged patient follow up due to the risk of mucosal metastasis after apparent successful treatment [13,14].*Leishmania (V*.*) shawi* was readily detected by the RPA-LF test. Early studies suggested that *L*. *shawi* was not frequently reported in humans and seemed to be of low prevalence in nature [15,16]. However, more recent studies in Northeastern Brazil found that 6.5% (5/77) of isolates were identified as *L*. *shawi* and that some of them could be considered hybrids with *L*. *braziliensis* [17].The RPA-LF was less efficient at amplifying *L*. *naiffi*, a species found in armadillos and occasionally infecting humans in different countries of South America [18,19]. Therefore, further test optimization would be necessary for epidemiological studies aimed at this particular species.

During the development phase, we detected variability in distinct batches of the lateral flow strips (Milenia Biotec, Germany) regarding increased background that led to the appearance of faint test bands in the negative controls. The problem was resolved by using higher dilutions of the amplification product (1:100–1:200). Each laboratory should standardize and select the lateral flow strips that best suits its needs. There are different commercial options of immunochromatographic strips for lateral flow reading. They are offered in containers with multiple strips (Milenia, Biotec), individual cards (Abingdon Health, UK), or cassettes (UStar, China) that are putatively less prone to contamination.

Scrapings or brushings of cutaneous lesions absorbed in filter paper were shown to be amenable to molecular diagnosis using PCR [20]. We have already shown that RPA-LF could use this preservation-transportation method to amplify *Leishmania* DNA from the blood of dogs infected with *L*. *chagasi* [8].The test was capable of amplifying DNA equivalent to 0.1 parasites in the reaction mix, which was comparable to the detection limit of our qPCR. Preliminary results using a small number of samples from lesions suggested that RPA-LF can efficiently detect parasite DNA in the presence of host DNA with high sensitivity and specificity. Nevertheless, the diagnostic sensitivity will have to be evaluated under field conditions in a larger number of patients. It is well established that parasite burdens tend to be highly variable and that parasites are more difficult to detect in chronic lesions [4]. Therefore, it will be particularly important to evaluate the diagnostic sensitivity of the RPA-LF in chronic lesions with >3 months of evolution.

A significant advantage of the RPA-LF is that samples can be rapidly processed, without the need of sophisticated equipment, outside of a traditional laboratory (e.g. at a house, school, or community center). Furthermore, initial evaluations strongly suggested that DNA extraction could be accomplished efficiently using a method that does not require equipment other than a boiling bath, giving additional support to the feasibility of adapting RPA-LF to the POC. RPA-LF is a less complex test than other isothermal amplification methods. RPA-LF results would be available in approximately one hour and the patients could initiate treatment if tested positive. Compared to a PCR reference test, this approach should enable earlier initiation of treatment, significantly increasing compliance and treatment efficacy. The need for delivering samples to a central reference lab, that leads to delayed therapeutic decisions and increased risk of patient loss, would be avoided. Importantly, the implementation of the field-applicable RPA-LF could replace or repurpose the need for experienced microscopists (and microscopes). It will improve the efficiency to diagnose leishmaniasis of short evolution time and, more importantly, in chronic lesions with parasite burdens below the microscopy threshold. The RPA-LF test may well fill the need for a field-applicable test, which is critical to cutaneous and mucosal leishmaniasis management.

## References

[1] Alvar J, Velez ID, Bern C, Herrero M, Desjeux P, et al (2012) Leishmaniasis worldwide and global estimates of its incidence. PLoS One 7: e35671 10.1371/journal.pone.0035671 22693548 PMC3365071

[2] Weigle K, Saravia NG (1996) Natural history, clinical evolution, and the host-parasite interaction in New World cutaneous leishmaniasis. Clinics in dermatology 14: 433–450. 8889321 10.1016/0738-081x(96)00036-3

[3] Blum J, Lockwood DN, Visser L, Harms G, Bailey MS, et al (2012) Local or systemic treatment for New World cutaneous leishmaniasis? Re-evaluating the evidence for the risk of mucosal leishmaniasis. Int Health 4: 153–163. 10.1016/j.inhe.2012.06.004 24029394

[4] Weigle KA, de Davalos M, Heredia P, Molineros R, Saravia NG, et al (1987) Diagnosis of cutaneous and mucocutaneous leishmaniasis in Colombia: a comparison of seven methods. Am J Trop Med Hyg 36: 489–496. 2437815 10.4269/ajtmh.1987.36.489

[5] Barroso-Freitas AP, Passos SR, Mouta-Confort E, Madeira MF, Schubach AO, et al (2009) Accuracy of an ELISA and indirect immunofluorescence for the laboratory diagnosis of American tegumentary leishmaniasis. Trans R Soc Trop Med Hyg 103: 383–389. 10.1016/j.trstmh.2008.12.019 19211118

[6] Romero LI, Paz HM, Ortega-Barria E, Bayard V, Hochberg LP, et al (2004) Evaluation of serological assays based on a novel excreted antigen preparation for the diagnosis of Cutaneous Leishmaniasis in Panama. J Microbiol Methods 57: 391–397. 15134886 10.1016/j.mimet.2004.02.008

[7] Adams ER, Gomez MA, Scheske L, Rios R, Marquez R, et al (2014) Sensitive diagnosis of cutaneous leishmaniasis by lesion swab sampling coupled to qPCR. Parasitology 141: 1891–1897. 10.1017/S0031182014001280 25111885 PMC4654403

[8] Castellanos-Gonzalez A, Saldarriaga OA, Tartaglino L, Gacek R, Temple E, et al (2015) A Novel Molecular Test to Diagnose Canine Visceral Leishmaniasis at the Point of Care. The American journal of tropical medicine and hygiene: 15–0145. 10.4269/ajtmh.15-0145 PMC4703275 26240156

[9] Piepenburg O, Williams CH, Stemple DL, Armes NA (2006) DNA detection using recombination proteins. PLoS Biol 4: e204 16756388 10.1371/journal.pbio.0040204 PMC1475771

[10] Pita-Pereira D, Lins R, Oliveira MP, Lima RB, Pereira BA, et al (2012) SYBR Green-based real-time PCR targeting kinetoplast DNA can be used to discriminate between the main etiologic agents of Brazilian cutaneous and visceral leishmaniases. Parasit Vectors 5: 15 10.1186/1756-3305-5-15 22240199 PMC3274473

[11] Harris E, Kropp G, Belli A, Rodriguez B, Agabian N (1998) Single-step multiplex PCR assay for characterization of New World Leishmania complexes. Journal of Clinical Microbiology 36: 1989–1995. 9650950 10.1128/jcm.36.7.1989-1995.1998 PMC104966

[12] Organization WH (2010) Control of the leishmaniases. World Health Organization technical report series: xii. 21485694

[13] Arevalo J, Ramirez L, Adaui V, Zimic M, Tulliano G, et al (2007) Influence of Leishmania (Viannia) species on the response to antimonial treatment in patients with American tegumentary leishmaniasis. J Infect Dis 195: 1846–1851. 17492601 10.1086/518041

[14] Llanos-Cuentas A, Tulliano G, Araujo-Castillo R, Miranda-Verastegui C, Santamaria-Castrellon G, et al (2008) Clinical and parasite species risk factors for pentavalent antimonial treatment failure in cutaneous leishmaniasis in Peru. Clin Infect Dis 46: 223–231. 10.1086/524042 18171254

[15] Lainson R, Braga R, De Souza A, Povoa M, Ishikawa E, et al (1989) Leishmania (Viannia) shawi sp. n., a parasite of monkeys, sloths and procyonids in Amazonian Brazil. Annales de parasitologie humaine et comparée 64: 200 2504099 10.1051/parasite/1989643200

[16] Shaw J, Ishikawa E, Lainson R, Braga R, Silveira F (1990) Cutaneous leishmaniasis of man due to Leishmania (Viannia) shawi Lainson, de Souza, Povoa, Ishikawa & Silveira, in Para State, Brazil. Annales de parasitologie humaine et comparée 66: 243–246. 10.1051/parasite/1991666243 1822654

[17] Brito ME, Andrade MS, Mendonca MG, Silva CJ, Almeida EL, et al (2009) Species diversity of Leishmania (Viannia) parasites circulating in an endemic area for cutaneous leishmaniasis located in the Atlantic rainforest region of northeastern Brazil. Trop Med Int Health 14: 1278–1286. 10.1111/j.1365-3156.2009.02361.x 19708899

[18] Naiff RD, Freitas RA, Naiff MF, Arias JR, Barrett TV, et al (1991) Epidemiological and nosological aspects of Leishmania naiffi Lainson & Shaw, 1989. Mem Inst Oswaldo Cruz 86: 317–321. 1842423 10.1590/s0074-02761991000300006

[19] Pratlong F, Deniau M, Darie H, Eichenlaub S, Proll S, et al (2002) Human cutaneous leishmaniasis caused by Leishmania naiffi is wide-spread in South America. Ann Trop Med Parasitol 96: 781–785. 12625932 10.1179/000349802125002293

[20] Veland N, Boggild AK, Valencia C, Valencia BM, Llanos-Cuentas A, et al (2012) Leishmania (Viannia) species identification on clinical samples from cutaneous leishmaniasis patients in Peru: assessment of a molecular stepwise approach. Journal of clinical microbiology 50: 495–498. 10.1128/JCM.05061-11 22116151 PMC3264178

